# A QTL on rat chromosome 7 modulates prepulse inhibition, a neuro-behavioral trait of ADHD, in a Lewis x SHR intercross

**DOI:** 10.1186/1744-9081-2-21

**Published:** 2006-06-12

**Authors:** Leandro Franco Vendruscolo, Elena Terenina-Rigaldie, Frantz Raba, André Ramos, Reinaldo Naoto Takahashi, Pierre Mormède

**Affiliations:** 1 Laboratoire de Neurogénétique et Stress, UMR 1243 INRA – Université Victor Segalen, Bordeaux 2, Institut François Magendie, Bordeaux, France; 2 Departamento de Biologia Celular, Embriologia e Genética, Universidade Federal de Santa Catarina, Florianópolis, SC, Brazil; 3 Departamento de Farmacologia, Universidade Federal de Santa Catarina, Florianópolis, SC, Brazil

## Abstract

**Background:**

Attention deficit hyperactivity disorder (ADHD) is a complex neuropsychiatric disorder with a substantial genetic component. The Spontaneously Hypertensive Rats (SHR), considered as a good animal model of ADHD, also show less anxiety-like behaviors than Lewis (LEW) rats. The use of these inbred rat strains led us to the mapping of two quantitative trait loci (QTL), named *Ofil1 *(on chromosome 4) and *Ofil2 *(on chromosome 7), related to locomotion in the central and aversive area of an open field. Herein, we examined whether LEW and SHR rats differ in the acoustic startle reflex, a test used to study the neurobiology of anxiety, and in the prepulse inhibition of the startle response, which is known to be impaired in ADHD patients. The effect of the two aforementioned loci on these behavioral responses was also studied.

**Methods:**

For this latter purpose, rats deriving from an F2 intercross between the LEW and SHR strains were selected according to their genotype at markers flanking the QTLs and bred to obtain lines of rats homozygous LEW/LEW or SHR/SHR for each of the two loci, thus generating 4 genotypic combinations.

**Results:**

The SHR rats displayed decreased startle and prepulse inhibition levels when compared to LEW rats. *Ofil2 *affected prepulse inhibition in female rats only.

**Conclusion:**

The results suggest that the LEW and SHR strains are appropriate for studying mechanisms of sensorimotor gating and indicate that the locus *Ofil2 *on rat chromosome 7 contain genes controlling prepulse inhibition, a neuro-behavioral trait of ADHD.

## 1- Introduction

Attention deficit hyperactivity disorder (ADHD) is a complex neuropsychiatric disorder that includes behavioral and cognitive features such as inattention and impulsivity/hyperactivity. There is evidence of impaired cognitive control processes [1] and poor inhibition [2] in ADHD. This heterogeneous syndrome has a substantial genetic component as demonstrated by family and twin studies and by the association of ADHD with polymorphisms of several genes [3,4]. Although ADHD has been perceived as being more frequent among boys than among girls, there is evidence that in adulthood more females suffer from the disorder than males [5].

In preclinical research, one of the most extensively evaluated animal models of ADHD is the Spontaneously Hypertensive Rat (SHR). SHR rats, compared with their normotensive controls (WKY) and with other rat strains, show sustained attention deficit, hyperactivity in some situations, motor impulsiveness, are novelty seekers and risk-takers, thus featuring the main symptoms of ADHD [for review see [6,7]]. The SHR strain, besides being used in the study of ADHD, also provides an interesting tool to study anxiety-related traits. Compared to the Lewis (LEW) strain, SHR rats show higher levels of approach towards aversive areas in several behavioral paradigms such as the elevated plus-maze, open-field and black/white box, without differing in general locomotion [8-10]. However, they do not differ in the social interaction [8] and predator odor tests of anxiety (unpublished data). Therefore, the behavioral contrast between LEW and SHR rats appears to be limited to anxiety tests based on approach/avoidance tendencies. In this context, it could be hypothesized that these strain differences are related to their ability to refrain motor activity or impulsive behavior.

In order to investigate the molecular bases of the behavioral differences between LEW and SHR rats, a genome-wide quantitative trait locus (QTL) search using an intercross between these strains was performed [11]. Two female-specific QTLs, named *Ofil1 *(on chromosome 4) and *Ofil2 *(on chromosome 7), were found to be linked to locomotor activity in the center of the open field. In a subsequent study [12], we found that these two loci combined affected also prepulse inhibition (PPI) responses, with animals that showed higher central locomotion in the open field also displaying decreased levels of prepulse inhibition.

The open field was originally developed as a test of emotionality [13,14] and is generally considered to be a stressful, fear-arousing environment. More anxious, emotional animals tend to ambulate less and stay away from the central part of the arena in such conditions. It is also assumed that locomotor activity in such inescapable arena reflects the rewarding component of novelty [15]. The PPI test, in which an acoustic startle response is reduced by a prepulse stimulus, is considered an operational measure of sensorimotor gating [16]. Disruption of PPI has been associated with disorders of uncontrolled behavior such as schizophrenia and ADHD [17-19]. Moreover, PPI is reduced after administration of dopamine receptor agonists and normalized with antipsychotic drugs [17]. These effects have been interpreted as a result of the modulation of sensorimotor gating under conditions of enhanced exploratory behavior [20]. Studies indicate that structures contributing to PPI include the nucleus accumbens, hippocampus, amygdala and medial prefrontal cortex [21], which are known to control motivational and emotional states. Therefore, the genes influencing PPI and those influencing open field behavior are likely to be partially overlapping.

The objective of the present study was to compare for the first time, LEW and SHR rats of both sexes in the acoustic startle test, a paradigm used to study the neurobiology of fear and anxiety [22] and in the PPI test. In addition, we aimed to breakdown the influences of *Ofil1*, *Ofil2 *and their interaction on acoustic startle and PPI responses. For this latter purpose, rats deriving from an F2 intercross between LEW and SHR strains were selected for breeding based on their genotype at polymorphic markers flanking the two QTLs. Animals whose genotypes were homozygous LEW/LEW or SHR/SHR at either *Ofil1 *or *Ofil2 *were selected to produce an F3 generation with a known genotype at these loci only, the rest of the genome being a random assortment of alleles from one or the other founder strain. The phenotypic differences among these groups would therefore be the result of genetic variations within these chromosomal loci. A similar approach has been used previously with success [12,23,24]. Finally, blood pressure of all animals was also measured.

## 2- Materials and methods

### Animals

Male and female Lewis/CRLIFO (LEW) and SHR/CRL (SHR) rats were purchased from Charles River/IFFA CREDO. To obtain the F1 population, three LEW males were crossed with six SHR females and three SHR males were crossed with six LEW females. F1 rats were then inbred to produce the F2 generation. A total of 453 rats (F2) were selected based on polymorphic markers at *Ofil1 *(D4Wox22, 37.38 cM and D4Mgh6, 58.99 cM) and *Ofil2 *(D7Rat35, 6.83 cM and D7Mgh11, 2.30 cM). Animals that inherited the genotypes homozygous LEW/LEW (L) or SHR/SHR (S) at each locus (4 = *Ofil1 *and 7 = *Ofil2*) were used as founders (L4/L7, L4/S7, S4/L7 and S4/S7). Three to five breeder pairs for each genotypic line were used in the study and the litters were culled at 8 pups. To better delineate *Ofil1 *the animals belonging to the F3 generation were further genotyped with D4Rat61 (73.62 cM) and the rats were required to be homozygous at all three markers. Adult F3 rats (10-week old) of the four new lines were used in the behavioral tests. Body weight and number of rats (in parentheses) per group were: 353.1 ± 9.8 g (11) and 212.6 ± 4.4 g (14) for L4/L7 line; 297.0 ± 12.5 g (5) and 178 ± 7.3 g (5) for L4/S7 line; 332.4 ± 12.0 g (8) and 215.0 ± 6.3 g (5) for S4/L7 line; 328.3 ± 4.0 g (16) and 202.2 ± 2.2 g (12) for S4/S7 line; males and females, respectively). Additional groups of LEW (males: 298.7 ± 6.1 g (10) and females: 198.3 ± 1.7 g (10)) and SHR (males: 273.2 ± 4.1 g (6) and females: 169.5 ± 3.4 g (10)) rats at the same age were concurrently tested. All animals were kept in collective plastic cages (2–4 rats/cage) having food and water available ad libitum and maintained in a room with controlled temperature (21 ± 2°C), under a 12L:12D cycle (lights on at 07:00 hours). All procedures used in the present study complied with the "Principles of laboratory animal care" from NIH.

### Genotyping

Primers for microsatellite markers were purchased from Eurogentec (Seraing, Belgium), or from Research Genetics (Huntsville, USA). Genomic DNA was extracted from tail tissue using a commercial kit (Promega, Charbonnières, France). Genotype determinations were performed by polymerase chain reaction (PCR). In a 20-μl reaction volume, 50-ng of genomic DNA was mixed with 5-pmol of each primer and 0.4 U of Taq DNA polymerase (Promega, Charbonnières, France) in Promega type A buffer. Amplification was performed in microtitre plates on a Hybaid OmniGene thermocycler (Hybaid Limited, Teddington, UK). The PCR conditions were: initial denaturation at 96°C for 5 min followed by 35 cycles of 92°C for 40 s, 55°C for 1 min and 72°C for 30 s and one cycle at 72°C for 2 min. Alleles were separated on 3% agarose gels and visualized with ethidium bromide staining under ultraviolet light.

### Acoustic startle reflex

The acoustic startle chamber (Imetronic, Pessac, France) consisted of a transparent plexiglass cylinder of 24 cm diameter enclosing a plate mounted on a force sensor connected to a computer which recorded the force produced by the animal located on the plate. All acoustic stimuli were presented via a speaker mounted 31 cm above the plate. The chamber was located into an insulating enclosure. Animals were placed into the chamber under low illumination (40 lux) and under a continuous 70-dB white noise background. Following a 5-min acclimatization period, 50 acoustic trials (105 dB; 5 KHz; 40 msec) were presented. The inter-trial interval was randomly fixed between 20 and 40 sec. Startle amplitude was measured during 300 msec after startle pulse onset. For each rat, startle amplitude (in Newton) was averaged over 10 trials in 5 blocks (i.e. 50 trials).

### Prepulse Inhibition (PPI)

This test was carried out 1 week after the acoustic startle reflex test in the same apparatus, under low illumination (40 lux) and under a continuous 70-dB white noise background. Following an acclimatization period (5 min), the animals were exposed to 10 acoustic trials (105 dB; 5 KHz; 40 msec) in order to habituate to the acoustic stimulus. These data were not exploited. Immediately after, a schedule of four conditions was performed: no prepulse (10 trials), 94 dB (10 trials), 98 dB (10 trials), or 102 dB (20 trials) prepulses (total of 50 trials). The duration of the prepulse was 40 msec (2 KHz frequency). These conditions were presented pseudo-randomly every 20 sec. The time interval between prepulse offset and pulse onset was 100 msec. Analysis was performed on the percentage of inhibition induced by each prepulse, calculated using the formula: Percent PPI = [100 - (100 * startle amplitude at prepulse trial)/(startle amplitude at startle pulse alone)]. PPI parameters were chosen based on preliminary studies.

### Blood pressure

Systolic blood pressure was measured at the tail of conscious rats by a non-invasive indirect method using a sphingomanometric system (Letica, Spain) as previously described [9,11]. The animals were placed in a cylindrical restrainer which was introduced through an opening into a warming box (38°C). The rat's tail was kept outside the box and a pulse signal transducer and tail cuff (connected to a central digital unit, LE 5000) were placed around the rat's tail. The animals were left undisturbed for a minimum of 30 min, once a constant pulse signal was detected, 5-8 recordings of blood pressure were obtained and the average was calculated.

### Statistical analysis

All analyses were performed separately for males and females. For comparisons between LEW and SHR strains, startle amplitude and PPI data were analyzed by a two-way ANCOVA (strain vs. repeated measures). Blood pressure data were analyzed by a Student's t test. To specifically analyze the influence of L or S genotypes at each QTL as well as their interaction on startle, PPI and blood pressure data, a two- or three-way ANCOVA (*Ofil1 *and *Ofil2 *factors) with or without repeated measures was carried out exclusively with the data of the four recombinant rat lines. Body weight was included as a co-variable in order to rule out the influence of body weight in the analyses of startle reflex and prepulse inhibition data. LSD test was used for post hoc comparisons of means when appropriate. The accepted level of significance for all tests was p < 0.05. Data are presented in the figures as means and SEM.

## 3- Results

**Figure 1:**
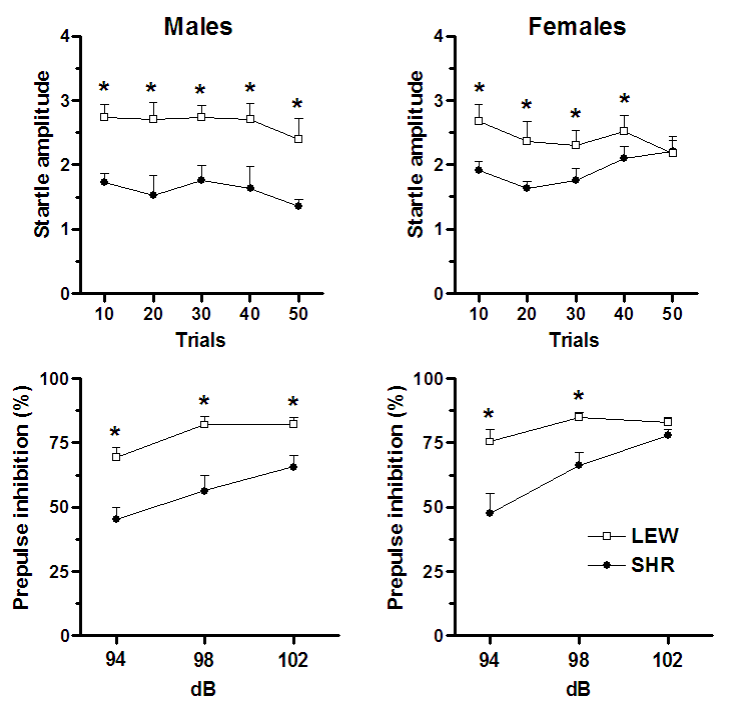
Acoustic startle (in Newtons, top panels) and percentage of prepulse inhibition of the startle reflex (bottom panels) at three prepulse intensities (94, 98 and 102 dB) displayed by LEW and SHR rats of both sexes. * Significantly different from SHR rats (p < 0.05).

Figure 1 illustrates the amplitude of the acoustic startle (top panels) and PPI (bottom panels) displayed by LEW and SHR rats of both sexes. For males, the two-way ANCOVA for repeated measures revealed an overall strain effect (F_(1,13) _= 4.68; p < 0.05) with LEW rats showing increased startle amplitude when compared with SHR rats. For females, the ANCOVA revealed a strain vs. repeated measure interaction (F_(4,72) _= 3.41; p < 0.013). Post-hoc comparisons indicated that LEW rats showed increased startle magnitude in relation to SHR rats in the first 40 acoustic trials (LSD, at least p < 0.03), but not in the latest 10 trials. Concerning PPI responses, the two-way ANCOVA for repeated measures in males revealed an overall effect of strain (F_(1,13) _= 15.27; p < 0.002) and of prepulse intensity (F_(2,28) _= 18.90; p < 0.001). LEW rats showed increased PPI levels in relation to SHR rats. For female LEW and SHR rats, the two-way ANCOVA revealed a significant strain vs. prepulse intensity interaction (F_(2,36) _= 4.76; p < 0.02). The post-hoc comparisons indicated that LEW rats showed increased PPI levels in relation to SHR rats at the two lowest prepulse intensities (94 and 98 dB; LSD, p < 0.002).

**Figure 2:**
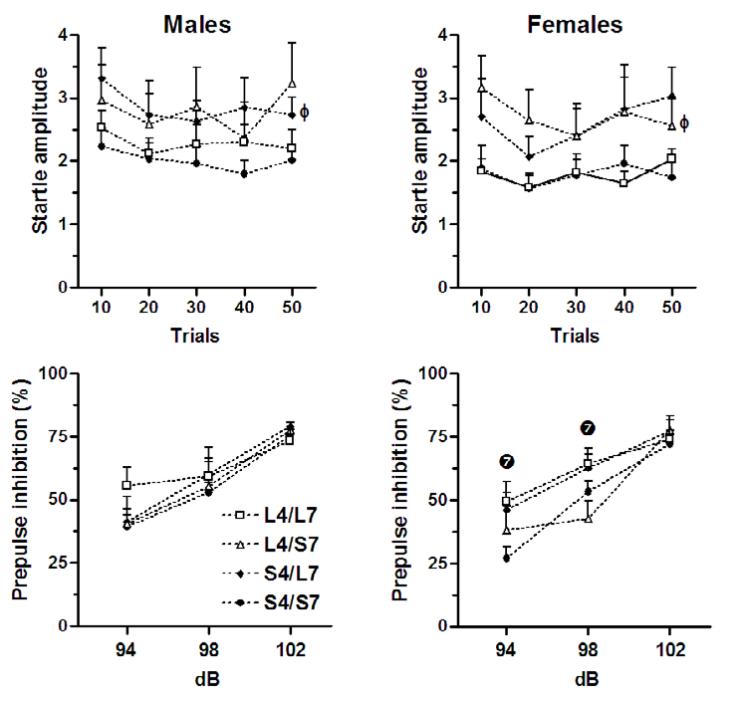
Acoustic startle (in Newtons, top panels) and percentage of prepulse inhibition of the startle reflex (bottom panels) at three prepulse intensities (94, 98 and 102 dB) displayed by F3 rats (according to line) of both sexes.  Indicates significant *Ofil2 *(on chromosome 7) effect (p < 0.05). Φ Significantly different from S4/S7 male rats or significantly different from L4/L7 female rats (p < 0.05).

Figure 2 illustrates the amplitude of the acoustic startle (top panels) and PPI (bottom panels) displayed by F3 rats (according to line) of both sexes. For males, the three-way ANCOVA revealed an interaction between the two loci (F_(1,35) _= 5.72; p < 0.023) on the magnitude of acoustic startle responses. The post-hoc comparisons indicated that the S4/L7 rats displayed increased startle responses when compared to S4/S7 rats (LSD, p < 0.04). For females, the three-way ANCOVA revealed a significant effect of repeated measure (F_(4,128) _= 4.51; p < 0.002) and a significant *Ofil1 *vs. *Ofil2 *interaction (F_(1,31) _= 10.18; p < 0.003), with the L4/S7 rats showing increased magnitude of the startle response when compared with L4/L7 rats (LSD, p < 0.05). In relation to PPI in males, the three-way ANCOVA (*Ofil1 *and *Ofil2*) for repeated measures revealed a significant effect of prepulse intensity only (F_(2,72) _= 47.28; p < 0.001), with the PPI increasing with prepulse intensity. For females, the three-way ANCOVA (*Ofil1 *and *Ofil2*) for repeated measures revealed a significant *Ofil2 *vs. prepulse intensity interaction (F_(2,64) _= 6.02; p < 0.004). An additional two-way ANCOVA performed separately for each prepulse intensity revealed a significant overall *Ofil2 *effect at 94 dB (F_(1,31) _= 4.16; p < 0.05) and 98 dB (F_(1,31) _= 7.51; p < 0.01) prepulse intensities, with the animals carrying the LEW alleles (i.e. L7) showing increased PPI levels in relation to the animals with the SHR alleles (i.e. S7).

For males, the Student's t test revealed that SHR rats displayed higher blood pressure than LEW rats (289.9 ± 6.9 vs. 238.7 ± 3.1 mmHg; t = 7.799; p < 0.001). Similarly, female SHR rats were hypertensive compared with female LEW rats (289.2 ± 5.1 vs. 236.7 ± 2.7 mmHg; t = 9.097; p < 0.001). Moreover, it was found that *Ofil1 *and *Ofil2 *did not affect systolic blood pressure in either males (mean of all F3 rats, 255.8 ± 2.5 mmHg) or females (mean of all F3 rats, 259.8 ± 2.8 mmHg).

## 4- Discussion

The present results of the acoustic startle reflex paradigm are in agreement with previous findings from other anxiety models [8-10], which indicated that SHR rats display less emotional reactivity than LEW rats. Moreover, SHR rats showed decreased prepulse inhibition when compared to LEW rats. The most important finding of the present study, however, was that the locus *Ofil2 *on rat chromosome 7 [11] affected significantly the PPI levels in female rats without affecting their startle responses. Furthermore, neither *Ofil1 *nor *Ofil*2 affected blood pressure thus confirming our previous studies [9,11].

The startle reflex, a fast twitch of the body musculature triggered by a sudden and intense acoustic stimulus, is frequently used to investigate the neurobiology of anxiety and fear [16,22]. The acoustic startle response is mediated by a relatively simple neuronal circuit located in the lower brainstem, but this primary pathway receives projections from higher structures that are known to control defensive behaviors [16,22]. In line with these observations, in the present study, LEW rats (considered as more "anxious") showed increased acoustic startle responses in relation to SHR rats (less "anxious"). Other studies have reported that SHR rats showed reduced startle response to an auditory stimulus when compared to most other strains [25,26]. It is noteworthy that whereas female LEW rats showed habituation in startle responses over acoustic trials the female SHRs did not habituate. Short-term habituation of startle is impaired in some neuropsychiatric disorders. For example, Braff et al. [27] reported that schizophrenic patients have extensive deficits in both PPI and acoustic startle habituation. Moreover, Grillon et al. [28] reported that startle magnitude was elevated in children with a parental history of anxiety disorder, whereas startle habituation and PPI were impaired in children with a parental history of alcoholism. Curiously, it has been reported that SHR rats show less anxiety-related behaviors [8-10] and higher alcohol intake than LEW rats [24,29].

In the PPI test, the startle magnitude is reduced when the acoustic stimulus is preceded by a non-startling prepulse and thereby is considered as an operational measure of sensorimotor gating [16]. In humans, impaired PPI has been associated with disorders of uncontrolled behavior, notably schizophrenia [17] and ADHD [18,19]. In rodents, deficits in PPI can be induced with the administration of various pharmacological agents, such as apomorphine, D-amphetamine and phencyclidine, and normalized by antipsychotic drugs [17]. We found that the SHR strain, considered as an animal model of ADHD [6,7], showed significantly decreased levels of PPI when compared to the LEW strain. However, other studies [26,30] reported that SHRs, compared to either Wistar-Kyoto or Sprague-Dawley rats, did not show any deficit in PPI. These findings, together with the present results, suggest that the LEW strain may show elevated levels of PPI in comparison not only with SHR but also with other strains. In any case, this behavioral contrast between LEW and SHR rats in PPI responses provides a useful tool to study the genetic mechanisms underlying sensorimotor gating. To our knowledge, no direct comparison between these two strains has been carried out previously. Since there is evidence that SHR rats may habituate more slowly to novel environments we cannot rule out this issue as a potential confounding factor in the behavioral differences observed between LEW and SHR rats.

It is puzzling that PPI-deficits can be induced by amphetamine in rodents and humans [21] while methylphenidate, another dopamine releaser drug, can ameliorate the symptoms of ADHD. Hawk et al. [19] recently reported that methylphenidate selectively increased PPI among boys with ADHD to a level comparable to that of controls. Very recently, Yamashita et al. [31] reported that a high dose of methylphenidate (60 mg/kg) significantly impaired PPI in wild-type mice but the same pharmacological treatment significantly reversed PPI deficits in dopamine transporter knockout mice. Thus, it should be of interest to test LEW and SHR rats treated with methylphenidate in the PPI test.

It is important to emphasize that SHR rats showed similar levels of startle reflex and PPI in response to either acoustic or tactile stimuli (airpuff) [30]. These findings suggest that the differential startle responses and PPI between LEW and SHR rats observed in the present study are more likely to be related to their behavioral reactivity rather than to acoustic acuity. Current opinion generally assumes that startle amplitude and PPI are independent variables that are under different genetic control [16,32]. Consistent with this idea, we found no statistical correlation between startle and PPI responses (data not shown), suggesting that the PPI differences are not likely produced by differences in startle magnitude.

Regarding the influence of *Ofil1 *and *Ofil2*, we have found previously [11] that they had a female-specific effect on central locomotion in the open field. The effect of *Ofil1 *was inverted as compared to the parental strains (i.e. LEW alleles promoted more instead of less central locomotion in the open field), whereas *Ofil2 *acted in the expected direction (i.e. LEW alleles reducing the trait). In a subsequent study, Mormède et al. [12] confirmed the role of these loci on behavioral responses by producing two rat lines with extreme genotypes for *Ofil1 *and *Ofil2*. It was found that the Low line (corresponding to S4/L7 rats in the present study) was less active than the High line (corresponding to the present L4/S7 rats) in the center of the open-field. This inhibition was not attributable to a classical "anxiety" factor as measured in the elevated plus-maze, in which the open-arm behaviors were not different between the lines. The High line also showed a deficit in PPI responses, suggesting that *Ofil1 *and *Ofil2*, which had been previously described as being related to anxiety, were indeed involved in sensorimotor gating mechanisms [12]. This study, however, did not allow us to sort apart the respective influences of these two loci because the effects of *Ofil1 *and *Ofil2 *were combined in only two rat lines. Recently, we have found that *Ofil1*, but not *Ofil2*, affected central locomotion in the open field in females [24]. Herein, it was found that females carrying two LEW alleles at *Ofil2 *showed increased PPI levels when compared to females carrying two SHR alleles at this locus. Therefore, it is possible to conclude that the PPI effects observed in the study by Mormède et al. [12] were likely produced by *Ofil2 *and not *Ofil1*. These findings further suggest that central locomotion in the open field and PPI responses are genetically dissociated in this specific model. It is important to note that the startle reflex was not affected by *Ofil2*. Furthermore, the PPI genetic effects reported herein are consistent with the profile of the parental strains, i.e. SHR alleles reducing the trait and LEW alleles increasing it. Finally, an interaction between *Ofil1 *and *Ofil2 *(i.e. epistatic effect) was also found for the acoustic startle reflex. The L4/S7 and S4/L7 lines displayed increased startle response in relation to L4/L7 and S4/S7 lines. These findings demonstrate the complexity of genetic influences on behavioral traits, probably involving gene-gene, gene-gender and gene-environment interactions.

Swerdlow et al. [33] reported that Sprague-Dawley rats displayed increased disruption of PPI responses by apomorphine, a dopaminergic agonist, when compared to Long Evans rats. Moreover, strain differences were observed in the efficacy of dopamine D2-like receptor-G-protein in specific brain areas. Correlational analysis revealed that in the striatum, cingulum, and cortex, greater dopamine-stimulated G-protein binding predicted less sensitivity to the PPI-disruptive effects of apomorphine [33]. As pointed by the authors, genes that regulate these differences may contribute to identification of heritable differences in patients with specific neuropsychiatric disorders. To date, only one study identified QTLs for PPI in rats. Palmer and collaborators [34] found a significant QTL on chromosome 2 and another suggestive QTL on chromosome 18. Furthermore, Joober et al. [35] reported several provisional QTLs specific for PPI on mouse chromosome 3, 5, 7 and 16. Very recently, Petryshen et al. [36] reported two QTLs on mouse chromosome 16. Therefore, the present study provides additional information by reporting a sex-specific QTL for PPI on rat chromosome 7. This region contains the Syn3 gene, which encodes Synapsin III (see ). Synapsins have been implicated in synaptogenesis and in the modulation of neurotransmitter release [37]. Since the human synapsin III gene is a potential locus for schizophrenia [38], we considered Syn3 as a candidate gene for *Ofil2*.

In conclusion, the present study confirmed the importance of sex and genetic factors on behavioral responses. The LEW and SHR strains, in addition to being useful in the study of anxiety-related behaviors, also seem appropriate for studying mechanisms of sensorimotor gating. Moreover, we identified a female-specific QTL on chromosome 7 that modulates PPI in rats, a neuro-behavioral trait found in ADHD (and in other psychiatric disorders). Further dissection of this locus should give information about sex and molecular mechanisms influencing disorders of uncontrolled behaviors and thereby open up new perspectives for neuropsychiatric therapies in humans.

## Competing interests

The author(s) declare that they have no competing interests.

## Authors' contributions

LFV carried out the data collection, performed the statistical analysis, drew the figures and wrote the manuscript. ETR and FR participated in the development of the four new rat lines though marker-assisted selection. AR, RNT and PM designed the study and the data analysis strategy, and participated in the interpretation of data and elaboration of the manuscript. All authors read and approved the final manuscript.
